# Neonatal–Maternal Bodyweight Ratio at Birth: An Indicator for First-Week Survival Prognosis in Canine Newborns

**DOI:** 10.3390/ani13213397

**Published:** 2023-11-01

**Authors:** Salvatore Alonge, Michela Beccaglia, Chiara Trovò, Monica Melandri, Giuseppe Migliaretti

**Affiliations:** 1 Società Veterinaria Il Melograno srl, Via Cavour 48, 21018 Sesto Calende, VA, Italy; drsalvatorealonge@gmail.com; 2 Post Graduate School of Medical Statistics & Biometry, University of Turin, Via Santena 5 bis, 10126 Torino, TO, Italy; 3 Ambulatorio Veterinario Beccaglia, Via Alessandro Volta, 2, 20851 Lissone, MB, Italy; ambulatoriobeccaglia@gmail.com (M.B.); trovo.vet@gmail.com (C.T.); 4 Department of Public Health and Pediatric Sciences, School of Medicine, University of Turin, 10126 Torino, TO, Italy; giuseppe.migliaretti@unito.it

**Keywords:** bodyweight, canine newborn, neonates, litter size, prognosis, survival

## Abstract

**Simple Summary:**

In dogs, neonatal bodyweight varies at birth and size-specific reference values are essential to correctly manage newborns. Present study aimed to define a new tool to evaluate neonatal weight, identifying puppies requiring intensive care. Records of 412 puppies, belonging to 89 litters of different size dogs, were retrospectively evaluated, recording litter size and gender, and considering neonatal mortality within the first week. The ratio between neonatal and maternal bodyweight was assessed in different litter size related to different size dogs, demonstrating that this parameter can be used to identify puppies at increased risk for one-week mortality and may be helpful in clinical practice, representing a suitable parameter to give a prognosis on 1-week survival immediately at birth.

**Abstract:**

**Objectives.** In dogs, neonatal bodyweight (NBW) varies at birth. Size-specific reference values for NBW are essential to correctly manage newborns. The present study aimed to define a new tool to evaluate NBW that could be routinely applied in canine neonatology, promptly identifying puppies requiring more care. **Methods.** The records of 89 litters were retrospectively evaluated. Data were grouped according to maternal bodyweight (MBW) in five categories: toy (≤5 kg), small (5.1–9.9 kg), medium (10–24.9 kg), large (25–39.9 kg), and giant (≥40 kg) dogs. At birth, the NBW of 412 puppies, alongside their litter size (LS) and gender (G), were recorded. Neonatal mortality within the first week was considered. The influence of MBW, LS, and G on NBW and the effect of NBW on the 1-week outcome were evaluated using ANOVA (*p* < 0.05). The ratio between NBW and MBW expressed as a percentage (N-MBW%), was assessed for each puppy. Through Receiver-Operating Characteristic (ROC) curves, N-MBW% thresholds between puppies alive or dead at one week of age were calculated in each group. **Results.** The LS was different among groups, except for small vs. medium dogs. In small litters, N-MBW% was lower in medium-, large- and giant-sized dogs than in toy and small dogs. In medium and large litters, N-MBW% differed among the five groups. Male and female N-MBW% differed among the five groups. Only in large and giant sizes did LS affect N-MBW%, which was lower in a large LS. The N-MBW% was higher in healthy puppies than in those who died within one week. The ROC-derived N-MBW% thresholds were as follows: 4.5% in toy-, 3.2% in small-, 1.5% in medium-, 1.2% in large-, and 1% in giant dogs. **Clinical significance.** The ROC-derived N-MBW% thresholds can be used to cautiously identify puppies at increased risk for one-week mortality and may be helpful in clinical practice, representing a suitable parameter to give a prognosis on 1-week survival immediately at birth.

## 1. Introduction

Neonatal health is affected not only by factors after birth but also during fetal life. Adequate growth as a fetus guarantees to the neonate that the maturity necessary at birth is attained alongside its ability to survive in extrauterine life [1]. Birthweight, which reflects intrauterine growth, is one of the most important determinants of neonatal survival [2,3,4].

In dogs, neonatal bodyweight (NBW) varies at birth [5]. Neonatal bodyweight is often compared to the bodyweight of adult subjects from the same breed: NBW is considered normal between 5 and 2.5% of adults’ bodyweight in small breeds, 2.5 and 1.7% in medium size, 1.7 and 1% in large and giant dogs [5].

Low birthweight neonates are considered a risk group for mortality [2,6,7]. Several factors have been identified to affect NBW [8]. First of all, the maternal bodyweight (MBW) was reported to influence NBW as heavier mothers usually give birth to heavier puppies [9]. The neonatal gender (G) must be considered, too, due to sexual dimorphism, which is already present at birth in some breeds, mainly in the giant ones, where female neonates are smaller than male ones [10]. Finally, the litter size (LS) should be taken into account, as larger litters usually comprise smaller neonates [11].

The literature referring to different species [12,13,14,15] reports that a low NBW is linked to a higher risk of death. The majority of papers studying critically low NBW consider the relationship between BW and short-term consequences, such as neonatal or pre-weaning mortality, a non-ambiguous and easy-to-quantify parameter that can be used as consensus outcome [16]. As low bodyweight has short- and long-term consequences on health, the early identification of affected newborns is recommended for appropriate management [16]. Size-specific reference values for NBW are essential to correctly manage newborns and promptly recognize puppies who are underweight at birth, possibly having a poorer outcome. In the canine species, which has the most extreme intra-specific size variability [17], several attempts have been made to express the concept of low NBW, which is vague in itself as well as in a huge size variability. Quartiles for NBW were calculated for single litters or breeds to identify underweight puppies [6,11], and breed-specific reference values for NBW were elaborated together with their related growth curves [9,10,17,18,19]. Unfortunately, all these cited methods were restrictive to very specific situations. Reference values for NBW referring to the size of different breeds were evaluated, too, but it was demonstrated that NBW varies among breeds of the same size [20]. Ideally, data regarding NBW should be breed-specific to account for the large heterogeneity in weight among dog breeds (from less than 2 kg to over 100 kg in adults), but there are almost 400 breeds of dogs, and for less common breeds, only small sample sizes are available [21]. It is certain that veterinary practitioners are in need of a tool to evaluate NBW, which should be easy to use, and applicable to every canine neonate, regardless of size, breed and litter.

Consolidated knowledge and recent updates on the studied topic summarized above were detected during our search procedure of the literature. The Pubmed database was searched between May 2018 and July 2023 with the following keywords: neonatal bodyweight, maternal bodyweight, birthweight, low birthweight, early neonatal mortality, neonatal mortality, early neonatal mortality rate, neonatal mortality rate, growth rate, growth curves, litter size, intra-uterine growth restriction. These keywords were applied first to all species, then using the filter “Other Animals” (thus excluding the human species) and finally summed to the keyword “dog” or “canine”. The following textbooks were consulted: *Veterinary Neonatology*, by Veronesi, C., Castagnetti, C., Taverne, M.A.M. (2012) and *Veterinary Reproduction and Obstetrics*, by Noakes, D.E., Parkinson, T.J., England, G.C.W. Finally, the Congress Proceedings of the following scientific societies were consulted from 2014 onwards: World Small Animal Veterinary Association (WSAVA), European Veterinary Society for Small Animal Reproduction (EVSSAR), and European Society for Domestic Animal Reproduction (ESDAR).

The main aim of the present study was to define a new tool to evaluate NBW that can be routinely applied in canine neonatology: a tool exploitable and able to identify those subjects below a definite threshold who require intensive care to face their increased risk of neonatal death. Factors influencing neonatal bodyweight were identified, the relationship between neonatal bodyweight and neonatal survival at one week of age was investigated, and finally, the cut-off value for N-MBW% to detect puppies at increased risk was calculated.

## 2. Materials and Methods

### 2.1. Ethics

This study was performed in accordance with ethical guidelines on animal welfare, and all procedures were carried out according to the Italian legislation on animal care (DL 116, 27/01/1992) and the European Guidelines on Animal Welfare (Directive 2010/63/EU). The owner’s informed consent for data collection was obtained.

### 2.2. Study Design and Animals

A retrospective study was performed. The data of litters reported in the clinical records database were selected when satisfying the following requisites:

– before mating, all bitches were proven healthy at a breeding soundness examination, including a thorough history and an ultrasonographic reproductive screening [22,23];
– at mating, all the bitches had a body condition score (BCS) of 5/9 [24] to allow a proper evaluation of their actual MBW. The BCS was evaluated by trained vets, following the same scale evaluation as suggested by Ahlstrøm and colleagues [24];
– irrespective of the type of delivery, puppies were born at term according to fetal biometry and were fully developed [25,26,27,28]. The parameters used to estimate the parturition term were the inner diameter of the chorionic cavity (ICC) twenty days after mating and the biparietal diameter (BP) in late pregnancy (around 40 days after mating);
– only data from uncomplicated pregnancies without any evidence of embryo or fetal loss and stillbirth were included and fetal well-being was verified by fetal heart rate [29] at the time of ultrasonographic examinations performed either for fetal biometry or at impending parturition term.

Puppies and litters were grouped according to the maternal bodyweight—recorded before the beginning of pregnancy (at mating)—in the following five size categories: toy (≤5 kg), small (5.1–9.9 kg), medium (10–24.9 kg), large (25–39.9 kg) and giant (≥40 kg) dogs [26,30]. 

The NBW of enrolled puppies was recorded immediately at birth before the first suckling, and their gender and litter size were noted by the researchers. The 1-week neonatal outcome was evaluated, classifying neonates as dead or alive at 1 week of age.

### 2.3. Statistical Analysis

Data obtained from clinical evaluations were reported on an Excel 2010 Office file spreadsheet, and mean values ± SD were calculated for each recorded parameter. For each study group (i.e., size category), the average litter size was calculated (the mean number of delivered puppies ± SD), and then LS was classified as small, normal, or large if the LS itself was below, in or above the range of its group, respectively. The ratio between neonatal and maternal bodyweight was assessed for each puppy and expressed as a percentage (N-MBW%).

The normality of data distribution was checked using the Shapiro–Wilk test; statistical analysis was performed via ANOVA. The possible influence of litter size and neonatal gender on the N-MBW% in different breed sizes (as a function of MBW) was evaluated by using one-way ANOVA and the Bonferroni test for purposes of multiple comparisons. 

The relationship between N-MBW% and neonatal outcome at 1 week of age was investigated. The final aim of the study was to evaluate the effectiveness of the newly proposed parameter (N-MBW%) to predict neonatal survival rate at 1 week (Gold standard, alive or dead at 1 week of age). In each size group, the N-MBW% threshold between puppies alive or dead at 1-week of age was calculated by Receiver-Operating Characteristic analysis: a web-based calculator for ROC curves [31]. For this purpose, the gold standard was set as the puppy’s status at 1 week of age: dead or alive. The area under the curve (AUC) was calculated and considered as a suitable, single-valued measure of accuracy when >0.7 and even of high accuracy when >0.9 [32]. Sensitivity (SE), specificity (SP), positive (LR+), and negative (LR-) likelihood ratios were calculated for the obtained threshold in each study group.

Results were considered significant at *p* < 0.05. The statistical analysis was performed with the online tool VassarStats: Website for Statistical Computation (http://vassarstats.net, Vassar College, New York, NY, USA).

## 3. Results

**Figure 1:**
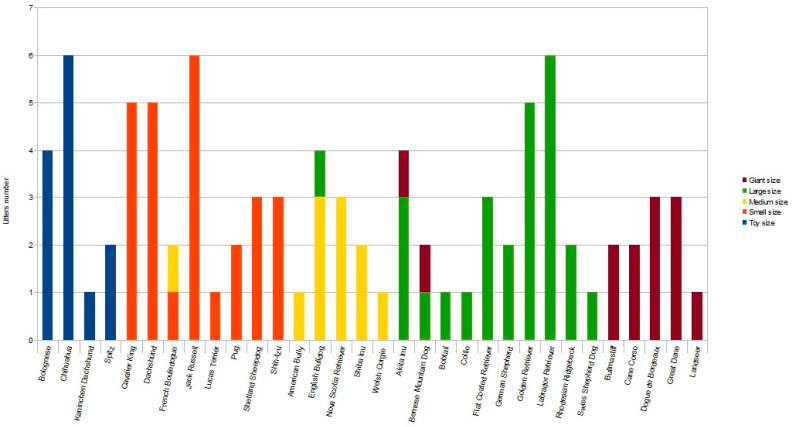
Histogram representing the number of litters for each breed enrolled in the different sized groups [toy (≤3 kg), small (3.1–9.9 kg), medium (10–24.9 kg), large (25–39.9 kg) and giant (≥40 kg) dogs] as indicated by different colors. Note that, depending on pre-gestational maternal bodyweight, some litters from the same breed were enrolled in two different study groups.

Following inclusion criteria, a total of 89 litters of different breeds were enrolled, allowing us to analyze the data of 412 puppies. The toy group included 36 puppies from 13 litters; the small group included 107 puppies from 26 litters; the medium group included 49 puppies from 11 litters; the large group included 143 puppies from 26 litters; the giant group included 77 puppies from 13 litters (Figure 1).

In toy-, small-, medium-, large- and giant-sized groups, the mean pre-gestational maternal bodyweight was 2.62 ± 0.39 kg, 6.55 ± 0.89 kg, 18.10 ± 4.85 kg, 31.79 ± 3.58 kg, and 56.47 ± 7.01 kg, respectively.

In toy-, small-, medium-, large- and giant-sized groups, the mean neonatal bodyweight was 0.14 ± 0.03 kg, 0.22 ± 0.05 kg, 0.32 ± 0.08 kg, 0.45 ± 0.07 kg, and 0.58 ± 0.11 kg, respectively.

The average litter size was 3.1 ± 0.8 puppies in the toy, 4.8 ± 1.7 in the small, 5.6 ± 2.4 in the medium, 7.6 ± 3.0 in the large, and 9.0 ± 3.1 in giant dogs. The size of the litter based on the number of puppies is summarized in Table 1. The LS was statistically different among groups except for small vs. medium dogs (Table 2). In small litters, the N-MBW% was statistically lower in medium-, large-, and giant-sized dogs than in toy and small dogs, without differences between small- and medium-sized puppies (Table 2). In medium and large litters, the N-MBW% was statistically different among the five groups (Table 2). Only in large- and giant-size dogs was the N-MBW% affected by the litter size, resulting lower in larger litters (Table 2).

The male and female N-MBW% was statistically different among the five groups (Table 3). Even if not statistically different (with a *p*-value of almost 0.5 in present the data), N-MBW% turned out to be lower in females in the toy, large- and giant-size dogs (Table 3).

The first-week mortality rate in the overall population was 7.52%. If considered per group, it was as follows: 5.50% in toy-, 10.28% in small-, 16.32% in medium-, 3.49% in large-, and 6.49% in giant puppies. The N-MBW% was statistically higher in healthy puppies than in those who died within one week from birth (Table 4). The difference was more marked in smaller than in bigger dogs, though it remained statistically significant in all five study groups (Table 4).

**Figure 2:**
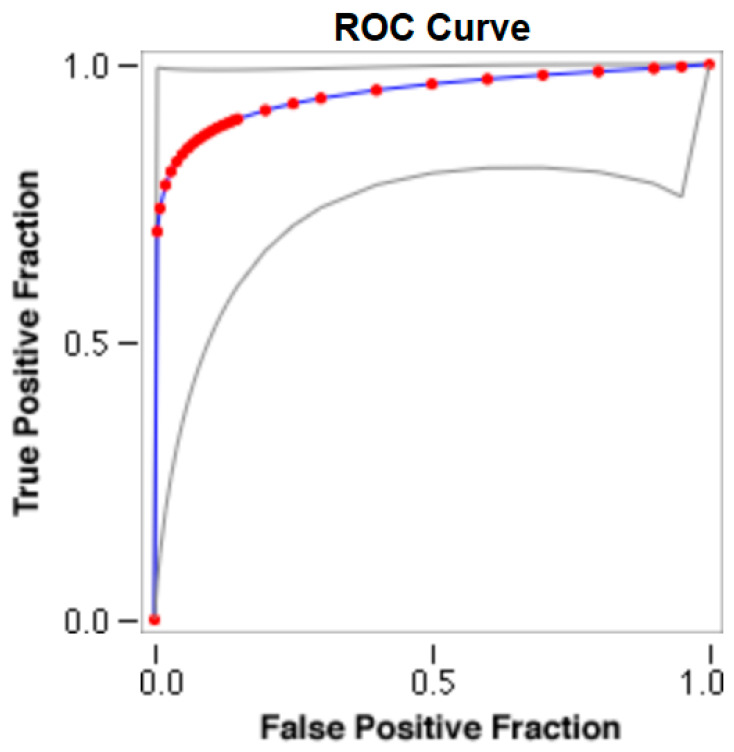
The cut-off for the N-MBW% threshold between puppies alive and dead at 1 week of age in the toy-size group from the web-based calculator for ROC curves.

**Figure 3:**
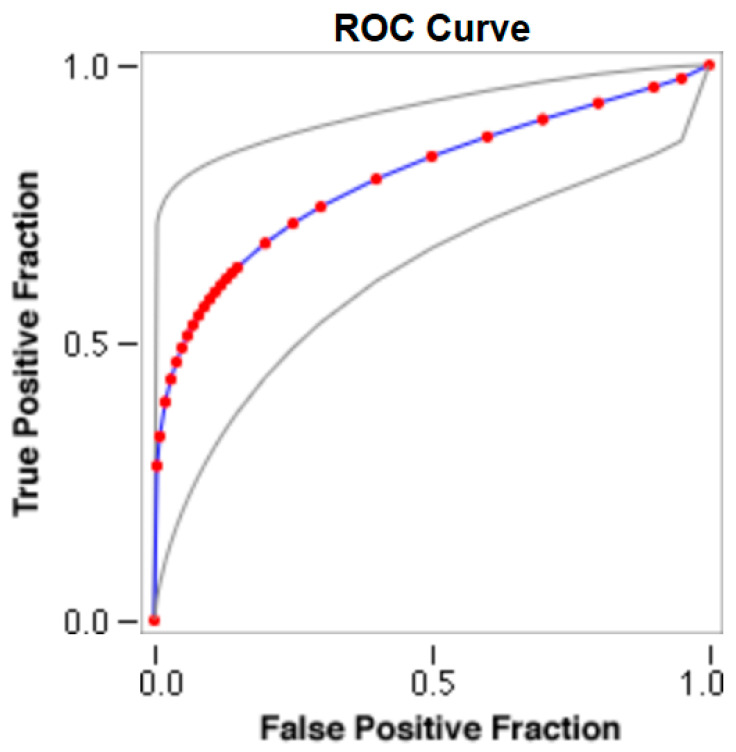
The cut-off for the N-MBW% threshold between puppies alive and dead at 1 week of age in the small-size group from the web-based calculator for ROC curves.

**Figure 4:**
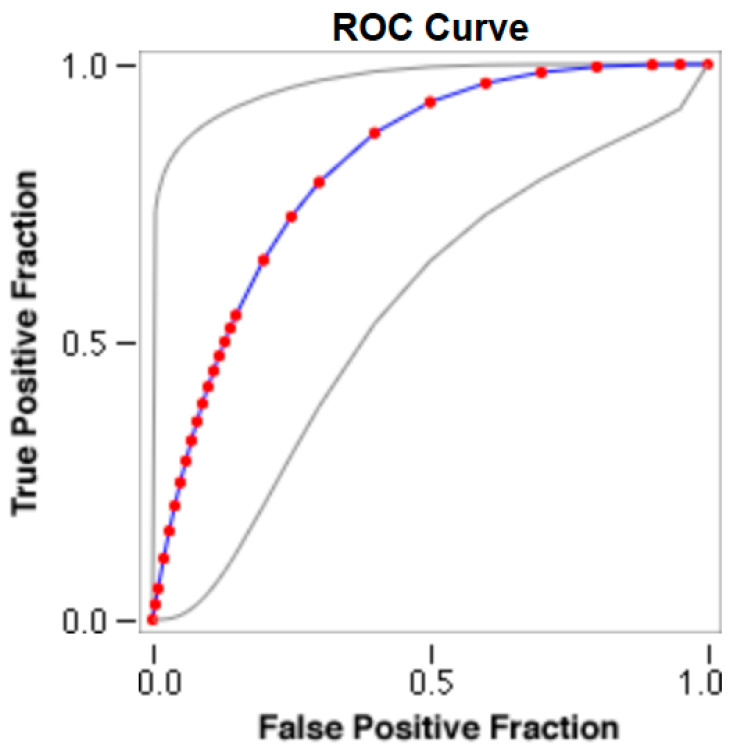
The cut-off for the N-MBW% threshold between puppies alive and dead at 1 week of age in the medium-size group from the web-based calculator for ROC curves.

**Figure 5:**
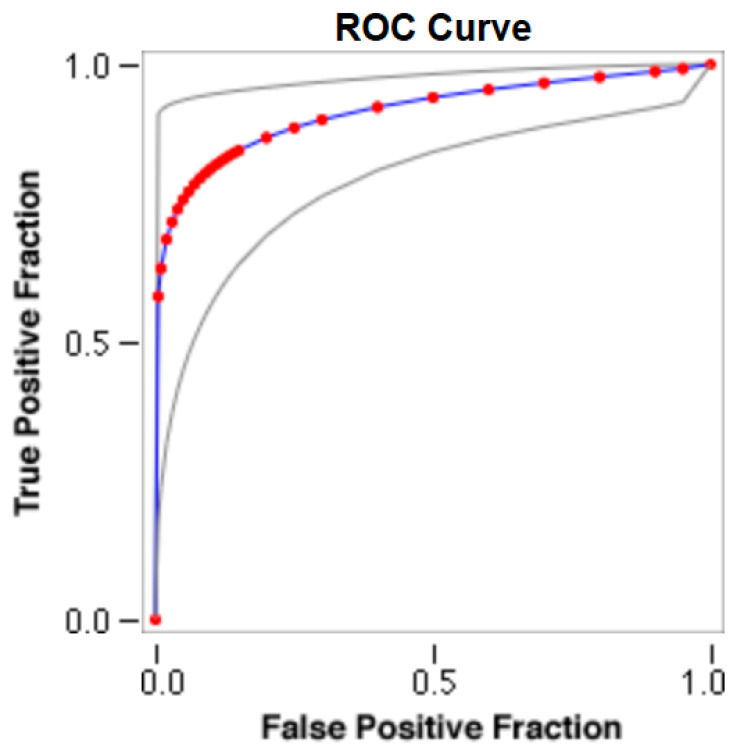
The cut-off for the N-MBW% threshold between puppies alive and dead at 1 week of age in the large-size group from the web-based calculator for ROC curves.

**Figure 6:**
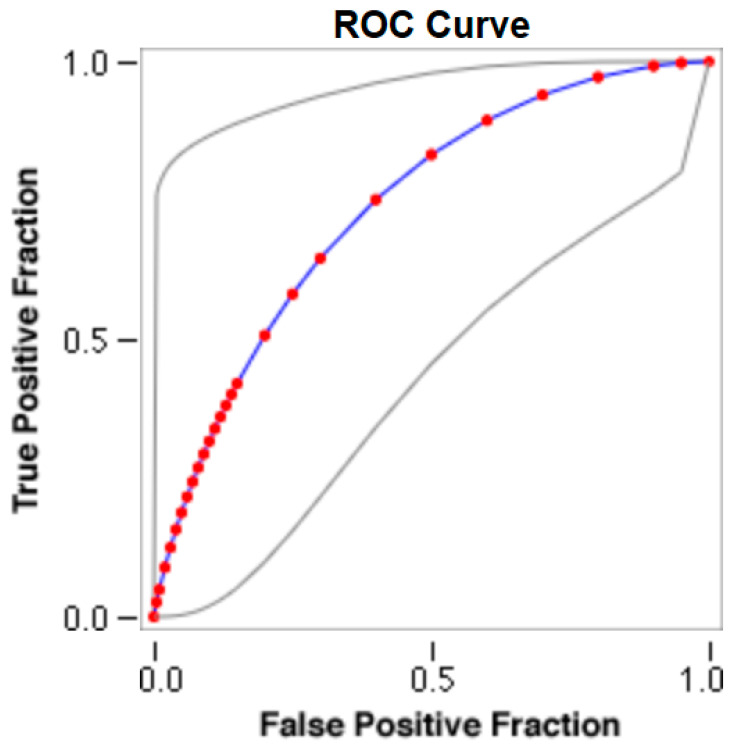
The cut-off for the N-MBW% threshold between puppies alive and dead at 1 week of age in the giant-size group from the web-based calculator for ROC curves.

The cut-offs for N-MBW% thresholds between puppies alive and dead at 1 week of age obtained from the ROC curves are reported for each size group in Table 5, together with their respective AUC (Area Under the Curve), sensitivity and specificity, and LR+ and LR− (Table 5, Figure 2, Figure 3, Figure 4, Figure 5 and Figure 6). The N-MBW% threshold between puppies at risk for death (below threshold) or health (over the threshold) at 7 days of age was 4.463% in toy (AUC 0.947), 3.218% in small (AUC 0.798), 1.545% in medium (AUC 0.816), 1.242 in large (AUC 0.916) and finally 0.979 in giant dogs (AUC 0.739).

## 4. Discussion

The results of the present study suggest that the one-week outcome is significantly conditioned by N-MBW%. The N-MBW% was statistically influenced by the dam size (reflecting the MBW) in all size categories; in large and giant dogs, it was statistically influenced by litter size. In the most extreme size categories, where sexual dimorphism was more enhanced [10], N-MBW% also showed a tendency to be affected in toy, large, and giant dogs as well as by gender. Finally, the N-MBW% was higher in healthy puppies than in those who died within one week in all size categories. 

The overall mortality rate reported during the first week of life in the present study (7.52%) was in line with previous reports by Tønnessen and colleagues (mortality rate: 8% within the first 8 days of life) [30] and Moon and colleagues (mortality rate within 7 days: 20% by Cesarean section and 25% by natural delivery) [33]. When considering the study groups, the mortality rate was below the mean for toy (5.50%), large (3.49%), and giant (6.49%) sizes and slightly over for the small (10.28%) size group. The first-week mortality rate was over the mean of the present study only for the medium-size group (16.32%); however, it still remained within the rates reported in the literature (20–25%, [33]). This is probably due to the presence in this same-size group of many puppies belonging to breeds at increased high risk for early neonatal mortality [30]: out of 49 puppies, 18 were English Bulldogs, 4 were French Bulldogs, and 4 were American Bullies. 

The N-MBW% can easily be applied to puppies of any size, independently of the breed, and even to mongrels relying on MBW only. It should be noted that some breeds (i.e., English Bulldog, French Bouledogue, Akita Inu, and Bernese Mountain Dog) were represented by litters classified in different size groups according to the MBW at mating time. This index overcomes the limits reported for quartiles, NBW reference ranges, and growth curves: all these parameters are highly specific for single breeds or even single litters and require elaborate mathematical calculations [2,6,10,17,18,20]. Instead, the N-MBW% can easily be calculated directly from MBW and NBW, and it is not plagued by breed issues; its categories only refer to the size, which is derived from the MBW.

To avoid possible BIAS, in the present study, only bitches with a BCS of 5/9 were enrolled [24]. These bitches represent the ideal to be reproduced, while over- and underweight females should be excluded, at least temporarily, in order to warrant their wellbeing. However, this index could also, theoretically, be used in clinical practice for bitches showing a BCS that lies over or under the optimum, as the literature offers very clear tables to commute high or low bodyweight into the ideal one by reducing or adding definite percentages of weight to the actual one [24]. Thus, the newly suggested index could also cautiously be applied to puppies born from under or overweight mothers after mathematically correcting the actual bodyweight of the mother into the ideal one and then calculating the ratio between the ideal maternal bodyweight and the neonatal one. It would be interesting to further investigate in practice the use of this new parameter in over- and underweight bitches, but it would be difficult to gather sufficient amounts of data as these kinds of females should not be reproduced.

The development of management strategies to optimize the fetal/neonatal outcome supports the ethical concept of the fetus/neonate as a patient [34]. Although in the case of birth complications, rapid veterinary intervention is warranted in order to minimize the development of maternal inanition and fetal vitality depression [35], veterinary supervision during the perinatal period is not always possible as patients are not always presented for care prior to/during parturition [36]. In these circumstances, the education of the owner regarding the whelping process and neonatal health monitoring is even important for positive health outcomes [36]. Practitioners, as well as breeders, need to quickly understand if a puppy appearing smaller than its littermates lies over or below the normal N-MBW% of its mother’s size. In the case of a large- or giant-sized mother, the variability depending on litter size should also be taken into account in the evaluation of the normal N-MBW%. A patient subjectively appearing smaller than its littermates could be a small puppy, however, lying over the N-MBW%, and its survival prognosis be favorable, or it could stand below the threshold, requiring intensive care to face its increased risk of neonatal death.

The risk of neonatal death is often related to metabolic issues. Therefore, it is important to pay attention to the prevention, identification, and treatment of disorders associated with the neonatal triad (hypothermia, hypoglycemia, and dehydration) [36]. The prompt identification of newborns requiring additional and tailored assistance is key to reducing perinatal loss in small animals [37]. Most of the time, this negative process starts (or develops) from a puppy of low bodyweight, especially when it is not promptly recognized. During their first hours of life, the thermoregulation mechanism of newborn puppies is deficient in anatomical and metabolic factors [38]. The shivering thermogenesis is absent for up to 6 days of life [39]. Recently, a positive correlation between the puppy’s weight and the puppy’s ability to achieve thermostability was demonstrated [38]. Moreover, canine neonates have a very low body fat content, i.e., around 1.3%, and derive their energy from glycogenolysis [40]. After birth, the glycogen stored in the liver and muscles declines rapidly [41], and gluconeogenesis is very limited due to the immaturity of the liver itself [42]. For all these reasons, puppies are very susceptible to hypoglycemia and hypothermia, which can easily drive them to death [43]. Particularly, underweight puppies have an increased ratio between their body surface and body mass; thus, all the described phenomena are more marked, with less stable glycemia, lower body temperature, and a reduced ability to suckle [20,44]. All these factors, taken together, increase the risk of mortality in low-birthweight puppies [15,45]. That can explain why, among the overall population of neonates, those showing an N-MBW% below the expected interval face an increased risk of mortality within their first week of life.

While the growth rate results from colostrum and milk assumptions, the NBW depends on the intra-uterine development [2]. Its deficit is named an intra-uterine growth restriction (IUGR) [2], which is a gestational disorder that is well studied in other species [45]. In piglets, IUGR does not result from the limited uterine space in very large litters but from placentation disorders, with the development of small placentas limiting oxygen and nutrient delivery from the sow to the fetus [13]. In human medicine, it is very well known that IUGR is caused by an insufficient presence of nutrients in the mother and by maternal vascular disorders in 35% of cases [12]. The IUGR leads to reduced NBW, which is linked to all the metabolic events described above. In the canine species, very little is still known about the physiopathology of the placental vasculature, and even less is known about its pathology. The placenta is essential for the development of the fetus, and its impaired function can lead to neonatal mortality [46,47]. Recently, it was remarked that the placenta has an influence on the weight of neonates, which is essential for its development in intra- and extrauterine life, even if more studies are required to better elucidate these questions [48]. As in humans, placental weight, the extension of the transfer zone, and the placental total vascular area correlate closely with puppies’ birthweight in normal pregnancies [49]. Thus, the problem of IUGR is far from being understood in dogs. Due to the actual scientific delay in understanding the etiology of IUGR in dogs, veterinary practitioners are not able yet to prevent its development, leading to low NBW in canine neonates. Nowadays, to face its consequences, the only clinical activity practitioners can perform is to diagnose its presence as soon as possible, which is to say immediately at birth. The present study demonstrates that the N-MBW% can be a useful tool in the early recognition of such disorders. This new suggested index can be used in dogs of any size. Using the results presented in Table 4, a transient limitation of this study can be highlighted referring to big- and giant-size puppies. In fact, even if a statistically significant difference is reported, referring to N-MBW% between alive and dead puppies of big- and giant-size, a misclassification of newborns can take place due to the overlapping of standard deviations. This is due to the fact that the N-MBW% is much lower in big- and giant-size groups than in toy-, small-, and medium-ones, as, proportionally, toy, small and medium puppies are already born heavier than big and giant ones, and the bodyweight of big and giant mothers exceeds that of respective puppies to a greater degree [5]. Further studies are warranted to limit this possible overlap; in particular, the standard deviation should be reduced by increasing the number of enrolled subjects in big- and giant-size groups. In the meantime, it would be interesting to compare on a larger sample the prediction performance of different methods available to date, including NBW thresholds, quartiles, and growth curves.

For the ROC curve in each size group, the cut-off for N-MBW% to detect puppies alive or dead at one week of age was calculated. In all groups, the area under the curve was >0.7; thus, this test can be considered adequate and clinically applicable in everyday practice according to the classification proposed for diagnostic tests [32]. As suggested by the high value of LR+, in all groups of different-sized dogs, it is strongly expected that puppies with neonatal bodyweight that ensure the level of N-MBW% will be over the threshold to survive the first week. Thus, in clinical practice, the application of this new suggested index, expressed as group-specific N-MBW% thresholds, could represent an easy-to-apply tool to cautiously identify those puppies lying below this threshold, showing a low NBW when compared to maternal pre-gestational bodyweight that require more intensive neonatal assistance because they are at high risk of non-survival at one week of age.

## 5. Conclusions

Nowadays, veterinary medicine is still unable to prevent and treat IUGR in canine species; thus, the problem of low birthweight puppies must be faced.

The parameter described in the present study, N-MBW%, may be helpful in clinical practice, possibly representing a suitable tool to provide a survival prognosis on puppies’ outcome within their first week of life, immediately at birth. It can easily be calculated for each puppy from NBW and MBW on-site on the first day of the neonatal check. It is advisable to give specific intensive care to neonates precociously identified according to this parameter to improve their prognosis. The N-MBW% thresholds to cautiously identify puppies at increased risk for one-week mortality are as follows: 4.5% in toy dogs, 3.2% in small dogs, 1.5% in medium dogs, 1.2% in large dogs, and 1% in giant dogs.

## Figures and Tables

**Table 1:** Classification of small, medium and large litter sizes in the five study groups.

Size	Toy Bitches	Small Bitches	Medium Bitches	Large Bitches	Giant Bitches
Small Litter (n. puppies/litter)	1	≤3	≤3	≤5	≤6
Medium Litter (n. puppies/litter)	2–3	4–6	4–8	6–10	7–11
Large Litter (n. puppies/litter)	≥4	≥7	≥9	≥11	≥12

**Table 2:** Percentage ratio of neonatal to maternal bodyweight at birth in toy (≤3 kg), small (3.1–9.9 kg), medium (10–24.9 kg), large (25–39.9 kg) and giant (≥40 kg) dogs in small, medium and large litters.

Size	Toy		Small		Medium	Large			Giant
Puppies	3.1 ± 0.8 ^a^		4.8 ± 1.7 ^b^		5.6 ± 2.4 ^b^	7.6 ± 3.0 ^c^			9.0 ± 3.1 ^d^
Mean litter-size	Puppies	Mean % ± SD	Puppies	Mean % ± SD	Puppies	Mean % ± SD	Puppies	Mean % ± SD	Puppies
Small	1	5.1 ± 0.1 ^a^	≤3	3.3 ± 0.6 ^b^	≤3	1.9 ± 0.6 ^bc^	≤5	1.7 ± 0.2 ^c†^	≤6
Medium	2–3	5.8 ± 1.1 ^a^	4–6	3.4 ± 0.7 ^b^	4–8	2.0 ± 0.5 ^c^	6–10	1.4 ± 0.2 ^d‡^	7–11
Large	≥4	4.7 ± 0.8 ^a^	≥7	3.3 ± 0.2 ^b^	≥9	1.5 ± 0.4^c^	≥11	1.3 ± 0.1 ^d§^	≥12

Different letter superscripts denote statistical differences within rows; different symbol superscripts (†, ‡, §) within columns.

**Table 3:** Percentage ratio of neonatal to maternal bodyweight at birth in toy (≤3 kg), small (3.1–9.9 kg), medium (10–24.9 kg), large (25–39.9 kg) and giant (≥40 kg) dogs in males and females.

Size	Toy	Small	Medium	Large	Giant	
	Mean% ± SD	Mean% ± SD	Mean% ± SD	Mean% ± SD	Mean% ± SD	*p*-Value
Males	5.6 ± 1.3 ^a^	3.3 ± 0.7 ^b^	2.0 ± 0.6 ^c^	1.5 ± 0.2 ^d^	1.1 ± 0.1 ^e^	0.001
Females	5.2 ± 0.8 ^a^	3.4 ± 0.9 ^b^	1.9 ± 0.5 ^c^	1.4 ± 0.2 ^d^	1.0 ± 0.1 ^e^	0.001
*p*-Value	0.052	0.15	0.197	0.059	0.058	

Different superscripts denote statistical differences within rows and columns.

**Table 4:** Percentage of ratio neonatal to maternal bodyweight at birth in toy (≤3 kg), small (3.1–9.9 kg), medium (10–24.9 kg), large (25–39.9 kg) and giant (≥40 kg) dogs in puppies alive or dead at 1 week of age.

Size	Toy	Small	Medium	Large	Giant	
	Mean% ± SD	Mean% ± SD	Mean% ± SD	Mean% ± SD	Mean% ± SD	*p*-Value
Alive	5.4 ± 1.1 ^a^	3.4 ± 0.8 ^b^	2.0 ± 0.5 ^c^	1.4 ± 0.3 ^d^	1.0 ± 0.1 ^e^	0.001
Dead	4.2 ± 0.1 ^A^	2.5 ± 0.6 ^B^	1.5 ± 0.5 ^C^	1.1 ± 0.2 ^D^	0.9 ± 0.2 ^E^	0.001
*p*-Value	0.016	0.003	0.010	0.020	0.037	

Different superscripts denote statistical differences within rows and columns.

**Table 5:** Thresholds for neonatal–maternal bodyweight ratio (N-MBW%) between puppies alive or dead at one week of age calculated in each group analyzing the respective ROC curves.

Size	AUC	N-MBW% Threshold	Sensitivity (%)	Specificity (%)	Likelihood Ratio +	Likelihood Ratio −
Toy	0.947	4.463	86.12	92.57	11.6	0.15
Small	0.798	3.218	63.08	85.47	4.43	0.43
Medium	0.816	1.545	82.03	66.81	2.47	0.27
Large	0.916	1.242	79.22	92.20	10.16	0.23
Giant	0.739	0.979	72.03	63.22	1.99	0.44

## Data Availability

The data presented in this study are available on reasonable request from the corresponding author.
